# Forensic Analysis of Commercial Inks by Laser-Induced Breakdown Spectroscopy (LIBS)

**DOI:** 10.3390/s20133744

**Published:** 2020-07-04

**Authors:** Flavio Cicconi, Violeta Lazic, Antonio Palucci, Ana Cristina Almeida Assis, Francesco Saverio Romolo

**Affiliations:** 1 Department of Chemistry, University of Bologna, Via Selmi 2, 40126 Bologna (BO), Italy; cicconi.flavio@gmail.com; 2 ENEA, Department FSN-TECFIS-DIM, Via E. Fermi 45, 00044 Frascati (RM), Italy; antonio.palucci@enea.it; 3 Laboratório de Polícia Científica da Polícia Judiciária, Rua Gomes Freire, 1169-007 Lisboa, Portugal; ana.assis@pj.pt; 4 Department of Law, University of Bergamo, Via Moroni 255, 24127 Bergamo, Italy; francescosaverio.romolo@unibg.it

**Keywords:** forensic, LIBS, laser spectroscopy, paper, ink, toner, deposition order, depth profiling, classification, questioned document

## Abstract

Laser-induced breakdown spectroscopy (LIBS) was tested for all of the relevant issues in forensic examinations of commercial inks, including classification of pen inks on one paper type and on different paper types, determination of the deposition order of layered inks, and analysis of signatures and toners on one questioned document. The scope of this work was to determine the potential of a single LIBS setup that is compatible with portable instruments for different types of ink analysis, rather than building a very large database for inks and papers. We identified up to seven metals characteristic for the examined inks, which allowed to fully discriminate all eight black inks on one type of printing paper. When the inks were tested on ten different papers, the correct classification rates for some of them were reduced for reasons thoroughly studied and explained. The replicated tests on three crossing points, each one involving a pair of blue or black inks, were successful in five cases out of six. In the test simulating documents of forensic interest (questioned documents), LIBS was able to correctly identify the differences in three inks used for signatures on one of the three pages and the use of different printing inks on each page of the document.

## 1. Introduction

The examination of inks and toners is very important in any criminal investigation involving documents. The most common forensic issues deal with correspondence among members of criminal organizations and alteration of original documents, for example a modified check amount, an added line on a contract, or a false signature on a will. Forensic studies of inks mainly regard their discrimination from one sample to another and determining the deposition order (writing sequence) of intersecting pen lines on paper. A comprehensive review of the analytical techniques used for examination of questioned documents was published by Calcerrada and García-Ruiz [1], which refers about inks from pens and printers, papers and studies on intersecting lines.

According to Denman et al., about of 80% of document analysis cases involve ballpoint pens [2], which contain organic soluble dyes, solvents, resins, and additives [3]. Dyes determine the ink’s color; solvents contribute to the homogenization of the ink; resins confer viscosity, lubricant properties, adhesion on paper, and durability to the ink; while additives improve the ink’s performance. A ballpoint ink dries almost immediately on contact with paper. On the other hand, rollerball pens contain a water-based liquid or gel and colored or carbon black pigments that are insoluble. Rollerball pen ink is less viscous and more easily absorbed by paper than oil-based ballpoint ink.

Commonly, the first approach in ink analysis is visual examination, which may solve simple and obvious cases in a non-destructive way. This is the preferred approach because it preserves the document’s integrity as evidence. For more complex cases, and given a similarity between many commercial inks, it is necessary to employ one or more analytical methods that could be grouped into three classes: separation techniques, mass spectrometry (MS), and other spectroscopy techniques [4]. The most employed technique in discrimination of ink samples is thin-layer chromatography (TLC) [5,6]. This technique has a high discrimination power of over 90%, but it is destructive and cannot provide analytical results for non-soluble inks. Mass spectrometry supplies molecular and elemental information, with a possibility to quantify both major and trace elements, in some cases making discrimination of samples more selective than separation techniques. For example, Alamilla et al. analyzed 21 blue pen inks by laser ablation inductively coupled plasma mass spectroscopy (LA-ICP-MS) and identified Li, V, Mn, Co, Ni, Cu, Zn, Zr, Sn, W, and Pb in inks. The measured signals from these elements were normalized on Mg, Ca, and Sr in order to subtract the contribution of the paper. The successive chemometric data treatment provided complete differentiation among different brands and a partial differentiation within pen inks from the same brand [7]. In another study, Trejos et al. used LA-ICP-MS on 200 ballpoint and gel pens of black color, achieving correct discrimination between pens from different brands and between pens from the same brand but different models in more than 98% of cases [8].

Among spectroscopic techniques, inductively coupled plasma atomic emission spectroscopy (ICP-AES) and inductively coupled plasma mass spectrometry (ICP-MS) are not used for routine analysis as they are very costly, require maintenance, and involve sample preparation. In fact, these techniques are often used when other instrument techniques that are simpler, faster, and less expensive cannot provide a definitive answer. Another review of the instrumental techniques used for characterization of inks is given in [9]; among these are vibrational spectroscopies, such as Raman [10] and infrared (IR) techniques. These two types of techniques are non-destructive, have imaging capability, are based on instruments that are simple to use, and do not require sample preparation. Vibrational spectroscopy techniques combined with chemometric data processing are routinely used in forensic examinations [11]. These techniques have the potential to identify the chemical structure of the functional groups present in inks, colorants, and resins, but their success in classification is limited due to the similar functional groups present within many inks. Certain inks show intense fluorescence that covers the Raman spectra; for example, in [12] it was reported that only approximately 50% of gel and ballpoint inks from a set of 80 multicolored pens showed readable Raman spectra. Detection of Raman signals also depends on the wavelength of the laser excitation, as demonstrated for pen inks [13,14] and inkjet-printed samples [15]. Studies of the Raman spectral variability of 190 blue pens of different ink types, brands, models, and batches showed slight variability among oil-based pens, resulting in difficulties in discriminating their brands, while in gel inks the classification of samples by brands was successful due to the different colorants used for the inks [16]. Compared to Raman techniques, IR radiation achieves deeper penetration, and when the focus is on the ink, interfering signals from underlaying paper occur. However, measurements using total reflectance-Fourier transform infrared spectroscopy (ATR-FTIR) for 100 pens (ballpoint, rollerball, and gel pens) from different brands on three types of paper resulted in high classification rates, except on recycled paper [17]. Similar results using ATR-FTIR were obtained for 57 blue ballpoint line strokes, where the correct classification rate was 97.9% [18]. In some cases, the differences in inks could not be identified by vibrational spectroscopy, as reported in [19], where the obtained discriminating rates among inks from blue and black pens analyzed by FTIR were only 73% and 80%, respectively. UV/visible spectroscopy allowed better discrimination for the given sample sets by achieving correct classification in 80% (blue inks) and 100% (black inks) of cases.

Analysis of printed texts is similar to analysis of handwriting. In inkjet printers, the ink is water-based and contains pigments, dyes, oils, resin, solvents, driers, plasticizers, and other additives, while laser printers employ toners containing finely ground powders mixed with polymers, colorants, external additives, and charge control agents [20]. The use of reflectance NIR and chemometrics allowed discrimination of ten black toners from four brands printed on different papers [21]. The colored toners were easier to distinguish by vibrational spectroscopy than the black ones and Raman spectroscopy achieved 95.6% discrimination among 23 colored inkjet printer inks [22]. Another study reported results of Raman spectroscopy on printing inks or toners, where the discrimination capability achieved among sample sets of the same color ranged from 75 to 94% for inkjet inks and from 0 to 86% for toners [23]. After development of spectral libraries, it was possible to correctly classify all the samples in a blind test on 45 black toners from 18 manufacturers by applying FTIR spectroscopy [24].

Laser-induced breakdown spectroscopy (LIBS) provides multi-elemental analysis of sample layers ablated by laser pulses, thus allowing depth profiling measurements. This technique is only microinvasive; it does not require sample preparation, it is rapid, cost effective, and different portable LIBS instruments are available on the market. All these facts promote LIBS as a technique to be used in forensic applications. In LIBS analysis of blue and black ballpoint pens, the obtained discrimination rate ranged between 99.8 and 100%, which was comparable with the more complex LA-ICP-MS approach [25]. Similar conclusions were reached by Trejos et al., who reported discrimination by LIBS among black gel inks and ballpoint pen inks between 96% and 99% [8]. Other research papers published about pen ink analysis by using LIBS [26,27,28,29] reported that the discrimination power depends on the experimental conditions and the data analysis procedure. LIBS analysis of printing inks also showed a high classification rate, which was 100% for black toners and 97.8% for black inks from inkjet printers [30]. High classification rates of printing inks and toners were also reported in other works [28,31,32]. Until now, only one paper reported LIBS analysis to study the order of deposition of inks [33] and it regarded only artistic prints. Here, the layering order was correctly determined for superposition of inks containing Faust lamp black and Gamblin bone pigments, and for inks colored by Charbonnel Ocean blue and Charbonnel Prussian blue. Considering the questioned documents commonly encountered in forensic casework, determination of the deposition order of inks still remains a challenging issue for various instrumental techniques [34,35,36,37,38].

The characteristics of a LIBS system determines its performance; thus, the datasets for sample classifications are specific for the instrument. In this work, we present the results of LIBS analysis of inks from commercial pens using a fixed experimental setup that is compatible with portable instruments. The aim was to test for the first time the same LIBS apparatus in three scenarios of interest for forensic examinations: characterization of pen inks on the same paper, characterization of pen inks on different kinds of paper, and determination of the deposition order of inks on paper.

## 2. Materials and Methods

### 2.1. Samples

The first set of samples consisted of inks from 14 commercial ballpoint pens (8 black, 4 blue, 1 red, 1 green; listed in Table 1) placed on one sheet of printing copy paper (Fabriano brand, 0.78 g/m^2^), hereafter called paper #3. The inks were deposited by drawing manually very close parallel lines inside an area measuring 1 × 1 cm^2^. Before starting the LIBS measurements, the inks were left to dry for at least four hours. Each of the 14 samples was probed at six points, distanced for at least 1.2 mm in order to exclude the area affected by laser-induced shock waves and eventual redeposition of material ablated from nearby examined points. At one sampling point, ten laser pulses were applied registering the spectra after each pulse in order to monitor the in-depth distribution of the inks.

The second set of samples consisted of inks from the same 8 black pens reported in Table 1, placed over ten different types of support, including printing papers, notebook papers, envelopes, and recycled papers. This set contained 80 different samples, with each one measured at six different points (480 measurements).

**Figure 1:**
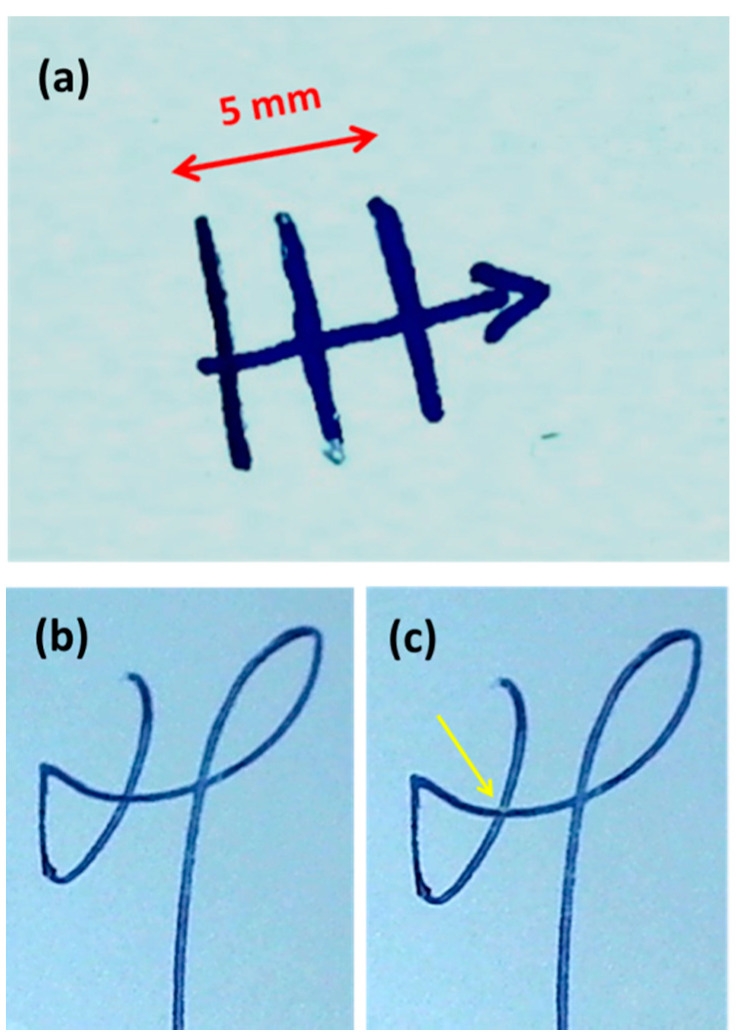
Photographs captured by the on-line camera of: (**a**) sample with crossed ink lines; (**b**) a signature on the contract before and (**c**) after the LIBS measurements. The yellow arrow indicates the ablated point.

The third sample set was provided by European COST Action CA16101 MULTI-FORESEE for a blind test and consisted of two pieces (replicates) of printing paper with four manually drawn lines by 3 ballpoint pens and 1 gel pen (Figure 1a), namely BIC Crystal, Pentel Super B, Staedther triplus ball, and Paper Mate pens. The line finishing with an arrow intersected the other three lines. The goals of the LIBS measurements were to characterize the four lines and to establish the chronological order of deposition of the inks at the intersections (crosses). One of the two pieces was exploited for the repeated (six) measurements of the inks and the blank paper, in addition to the single spot analysis of the cross points, while the second sample was probed at only one point per ink and per cross, with the aim of determining the margins of error in the ink classification process.

The fourth set of samples was provided by the Portuguese Police Forensic Laboratory as part of the COST Action CA16101 MULTI-FORESEE project for a blind test. It contained three printed pages with three signatures placed by different pens. The full specifications of this questioned document are given in Table 2. Example of the examined signatures before and after the LIBS measurement is shown in Figure 1b. In this case, the task was to verify if the same pen was used on all three pages (using a minimally invasive approach, i.e., by applying LIBS at only one point per signature) and if the printing toners were the same on all three pages. The toners were probed at six points by carefully choosing the sampling points in order to minimize the visibility of the laser-induced ablation.

### 2.2. LIBS Set-Up

The plasma was generated by an Nd:YAG laser (Quantel, CFR Ultra, Bozeman, MT, USA) emitting 6.5 ns pulses at 1064 nm. The laser energy was fixed to 30 mJ, and at each position ten laser pulses were delivered at repetition rate of 2 Hz. The beam was expanded x2.7 and then focused by a quartz lens f = 100 mm perpendicularly to a target. The sample’s support was mounted on a holder with three degrees of freedom, making it possible to adjust the height and keep the sample perpendicular to the beam over an area of about 10 cm^2^. The sample holder was placed on a motorized X-Y table and precise positioning on the sample spot was controlled by an HD camera [39].

The plasma emission was collected at angle of about 70° from the target plane by an optical system with a diameter 25.4 mm containing two quartz lenses with focal lengths of 75 and 150 mm. The light was focused on a 1-m-long fiber bundle containing four 600-µm-diameter quartz fibers that directed the signal to four compact spectrometers (Avantes AvaSpec-ULS2048L, Apeldoorn, Netherlands). The spectrometers cover the range of 200–795 nm and the spectral resolutions are 0.07. 0.09, 0.10, and 0.16 nm for intervals of 200–270, 270–400, 400–546, and 570–795 nm, respectively. The spectra were registered after each laser pulse by using the 0.5 µs acquisition delay and the minimum acquisition gate of 1.05 ms.

## 3. Results and Discussions

### 3.1. Analysis of Commercial Inks on One Paper Sheet

**Figure 2:**
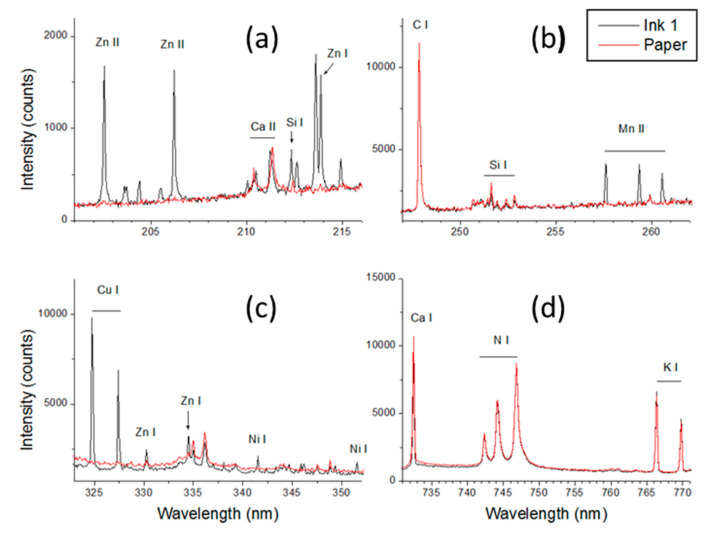
Characteristic spectral intervals after the first laser pulse from ink #1 and paper #1, averaged over six repeated measurements; in the top-left graph the unassigned lines belong to Cu II. (**a**): 201–216 nm; (**b**): 237–262 nm; (**c**): 322–353 nm; (**d**): 730–771 nm.

The first round of the measurements was performed on the 14 inks placed on the same sheet of printing paper, hereafter called paper #3, which was also characterized at six different points. An example of the spectra (characteristic intervals) taken on ink 1 and the blank paper is shown in Figure 2. The blank paper contained a high amount of Ca, which was visible from the many saturated lines. The high Ca content is due to the calcium carbonate (CaCO_3_) residue, which is commonly used as a paper filler. Paper fillers are mineral pigments that improve the surface properties of paper, such as the printability, and they affect the opacity, brightness, and color. Beside the elements from periodic groups I and II, the analyzed paper also contained Si and Al. In the spectra acquired on the inks, these elements had much weaker lines than on the blank paper, thus we did not consider their eventual presence in the examined ink. The analytical lines used for monitoring the elements characteristic for papers and inks are listed in Table 3, which were chosen carefully to be free of overlap, sufficiently intense for trace elements and not saturated for major elements (in paper). The atomic emission lines from nitrogen and oxygen were always observed in the spectra but were not considered further because they are present in the surrounding air. The detected overall plasma intensity was generally weaker on inks than on blank paper, which was also evident from the intensity of the plasma continuum above 300 nm (Figure 2). For this reason, we often observed an increase in the line intensities from ink components in the spectra produced by the second laser pulse (at fixed position), which mainly ablated the paper beside the residual ink layer. As the paper support is always involved in ablation, the absolute line intensities from elements belonging to an ink depend on its local thickness and distribution inside the laser spot. For this reason, in order to reduce the signal fluctuations from one point to another for one ink and to compare different inks, we applied the signal normalization on carbon because it is present in both papers and inks.

**Figure 3:**
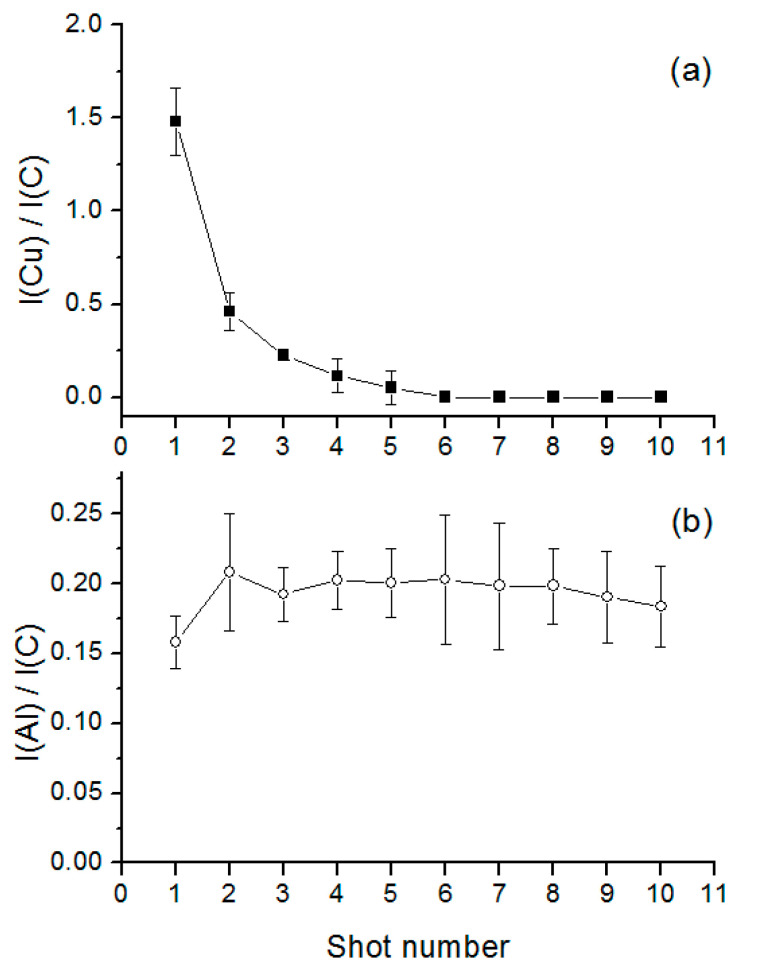
Average ratio of the Cu I (**a**) and Al I (**b**) line peaks normalized on the C I peak as a function of the shot number. The sample is ink #1 placed on printing paper #3.

The deposition of ink on paper does not produce a uniform layer and the penetration depth is expected to be irregular and dependent on the ink, the paper’s properties, and the way the writing tool is used on the paper. The elements characteristic for ink #1 and not present in the paper were Cu, Zn, Mn, Ni, Pb and Fe. The intensity of the emission lines from these elements rapidly decreased after the first laser pulse, as illustrated for the Cu spectral line (Figure 3a). Here, the signal from the Cu I line, which was the most intense among the element lines characteristic for this ink, completely disappeared after the first five laser pulses while its presence for pulses 2–5 could be attributed to the ink penetration into the paper. Laser ablation by the first pulse always involves paper below the ink layer because the line emissions characteristic for the blank paper are always visible, as for example Al I in Figure 3b. The error bars in Figure 3 represent the standard deviation calculated from six replicated measurements, where the signal variations could be also explained by different line widths and the applied pressures used in drawing them.

The elements that could be considered characteristic for the inks are only those not present in the paper or those producing signals (here measured through the ratio of the peak intensities from the analytical element line and C I line) which are higher after the first laser pulse than on the blank paper. In 14 examined inks we found seven characteristic elements, namely Cu, Zn, Ni, Mn, Pb, Fe, and Cr; their average line peaks normalized on the C I line, measured after the first laser pulse, are reported in Table 4. The relatively high fluctuations of the line intensity ratios from one sample are due to factors such as local variations of the ink thickness and paper coverage. Among eight analyzed black inks, we found one sample (ink #7) not containing any metal except traces of Mn, possibly indicating that the pigment was carbon black. All the other black inks contained Cu, except ink #4 where a large presence of Cr was found. The five analyzed blue inks, as well as the red and green ones, contained Cu and at least another two trace metals. Excluding ink #7 (black), the relative amount of Cu varied by more than ten times among the inks of the same color (black or blue).

The ink composition in ballpoint pens comprises a colorant, which is always a minor component of the ink (maximum 10% in weight). The most common dye for blue ballpoint pens is the phthalocyanine blue BN, a molecule where the transition metal that is coordinated inside the phthalocyanine ring is Cu. For this reason, Cu signals are the most characteristic trait for ink determination. The chemical complexity of the black inks is usually much higher because of the intrinsic difficulty in absorbing the entire visible wavelength. For this reason, black inks of commercial pens might contain not only a black pigment (for example lamp black, pigment black 11), but also various colorants of violet, blue, yellow, or even an intense red color [40,41]. Many black ballpoint pen inks also contain nigrosine, the main structural unit of which is phenazine. This mixture of synthetic black dyes is made by heating a mixture of nitrobenzene, aniline, and hydrochloric acid in the presence of copper or iron [41]. The other trace elements previously reported in some ink pigments analyzed by different techniques, such as Al, Ba, Li, Mo, Sn, V, and Zr [8,30], were not observed in our experiment, except for Al and Li, giving higher signals on blank paper than on the same paper covered by an ink.

**Figure 4:**
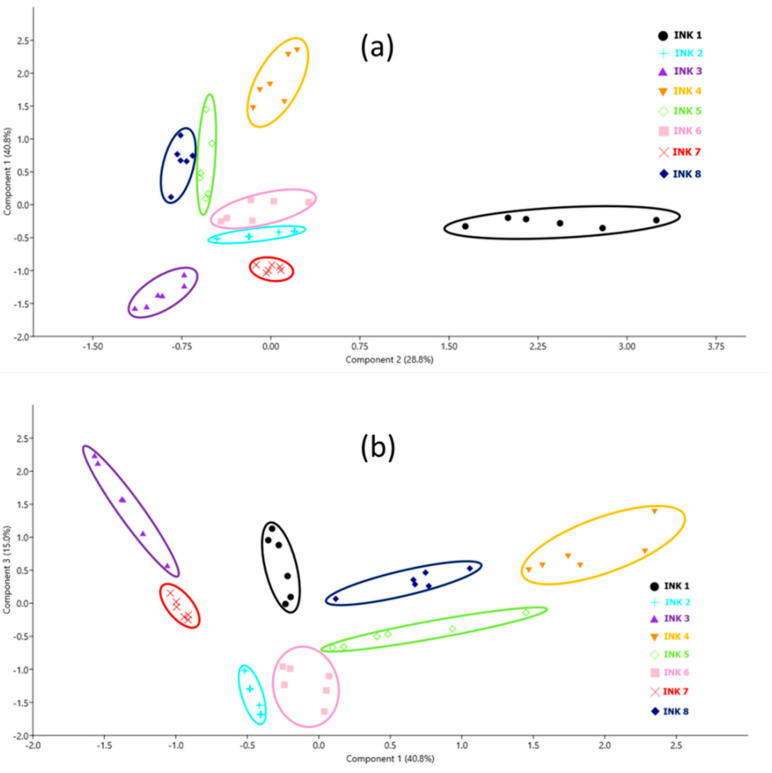
PCA plots for black inks over paper #3: (**a**) PCA1 vs. PCA2; (**b**) PCA3 vs. PCA1.

We also tested if the eight black inks on a single sheet of paper (#3) could be discriminated by applying principal component analysis (PCA), performed using Paleontological Statistics PAST 4 software (free access). The model was built using the peaks of Zn, Mn, Cu, Pb, Ni, Cr, and Fe normalized on the C I line (for the first laser pulse), and included 6 measurements for each of the 8 black inks. The obtained scores for the first three PCA components were 40.8%, 28.8%, and 15.0%, which together covered 84.6% of the variability. The PCA projections provided complete separation of the samples, as shown in Figure 4. We applied a linear discriminant analysis (LDA) classifier, where each data point is assigned to the ink group that gives the minimal Mahalanobis distance to the ink’s group mean. The examined black inks were 100% correctly classified, demonstrating the method’s capability to differentiate all of them when deposited on the same printing paper.

### 3.2. Analysis of Commercial Black Inks on Different Papers

There are many types of documents needing forensic examination, from ransom letters to printing papers, recycled or bleached papers, and others. With this perspective, the discrimination between various inks regardless of the paper type could be of great importance. Therefore, in our second sample set we tested inks from 8 black pens placed on 10 different paper types, including printing papers that normally contain whitening agents, fillers, and coatings; three letter bag papers, which might contain partially impermeable top coating; notebook paper; and recycled papers.

**Figure 5:**
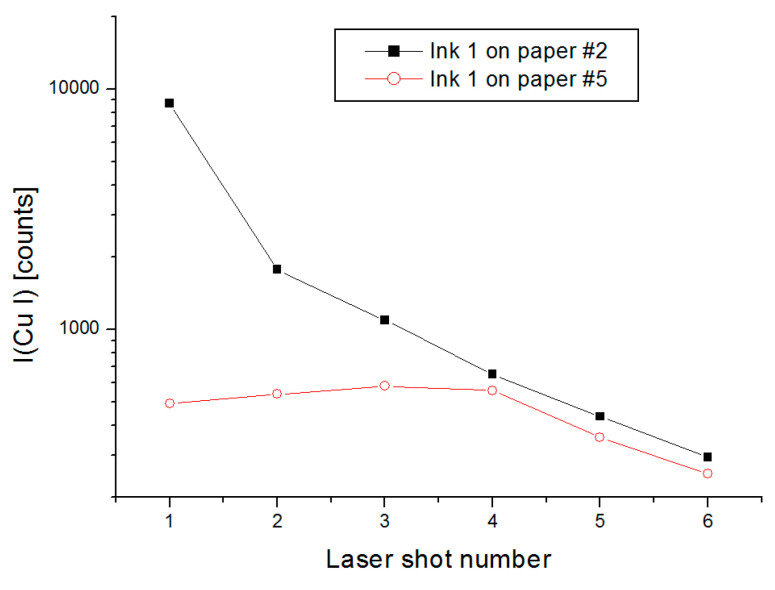
Intensity of the Cu I peak as a function of the laser pulse number for the ink #1 placed over printing paper #2 or recycled paper #5.

When passing from ink analysis on one type of substrate to those involving many types of paper, different variables must be taken into account. We showed that the paper is always ablated together with the ink, and that changes in the laser–paper interaction due to different optical, physical, and chemical properties contribute to the matrix effect. The papers themselves might contain some elements previously exploited for classification of the inks. In our tests, among seven elements used to distinguish the inks, Mn and Fe were found in eight papers from our sample set, but with weak line peaks compared to the inks containing these elements. The absorption of the ink also changed from one paper to another, whereby a higher absorption is equivalent to a lower amount of ink in the top layer ablated by the first laser pulse. An example of the influence of the paper absorption on the LIBS signal from one ink is shown in Figure 5. Here, the recycled paper is much more permeable to the ink than the printing paper, as evident from the almost constant Cu I emission (characteristic for the ink) during the first four laser pulses. Differently, on low absorbing printing paper the Cu signal is much higher after the first laser pulse than in the previous case, and it rapidly decays for the successive laser pulses applied at the same position. When comparing the Cu I line intensities normalized on the C I peak for the first laser pulse, in case of absorbing paper #5 the average measured value is 0.12 ± 0.02 while for the same ink on printing paper #2 it is about ten times higher, having the value of 1.1 ± 0.2.

**Figure 6:**
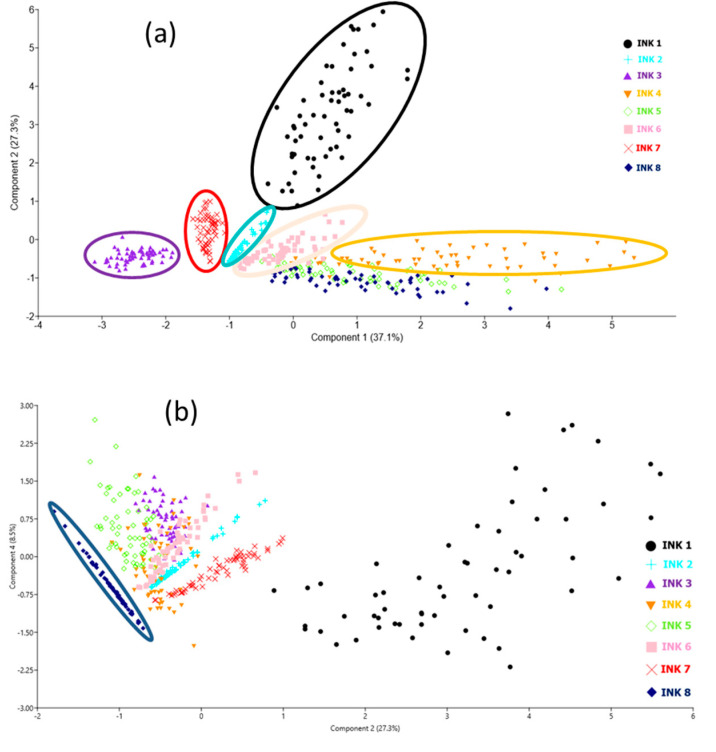
PCA plots for black inks over ten different papers: (**a**) PCA1 vs. PCA2; (**b**) PCA4 vs. PCA2.

We applied the PCA analysis for the data set containing a total of 480 data points from 8 black inks deposited on 10 different papers, where on each sample six measurements were taken. The model included seven elements from Table 4. Peaks were normalized on the C I peak and only the spectra acquired after the first laser pulse were considered. After applying the correlation analysis, the first three PCA components explained 80.6% of variables inside the data set, while the fourth PCA component covered 8.5% of variables. Compared to the plot shown in Figure 4a, the data relative to various papers show much greater scattering (Figure 6a). From this plot, it was already possible to completely separate inks 1, 2, 3, and 7. By examining various projections of the PCA components up to the fifth one, we found that plotting PCA 4 vs. PCA 2 separated ink 8 well, as shown in Figure 6b. On the one hand, inks #5 and #6 contain Cu, Zn, and Ni while ink #5 also has a small amount of Pb (see Table 4), which was not detected on some points, making difficult to differentiate it from ink #6, which does not contain this element. On the other hand, ink #4 contains the same metals as ink #5 but in higher amounts.

By applying LDA, we obtained the results reported in Table 5 as a confusion matrix, where off-diagonal counts indicate the number of points incorrectly attributed to another ink group. As expected from the PCA plots (Figure 6), inks 1, 2, 3, and 7 are 100% correctly classified. Ink 8 shows 10% erroneous attributions to inks 3 and 4, where ink 3 also contains Cr but at a much higher amount. Inks 4 and 8 differ in presence or absence of Ni and Cr (See Table 4), but the corresponding amounts of these elements are very low, which might explain the errors in classification. Inks 5 and 6 are correctly classified for about 80%. An interesting case is represented by ink 6, for which 20 out of 60 points were attributed to ink 2 although the latter should differ clearly due to the absence of Zn. The reason for this error was found in the three paper types containing some Zn on the surface, thus increasing the error rate for ink 6, which has only a small amount of this metal.

The correct classification rate of the examined black inks by applying LIBS and chemometrics was reduced from 100% to 89% (overall) when involving ten different paper types. In particular, failures were observed for inks that differed mainly in the contents of trace elements found also on some paper types. The LIBS technique provides elemental analysis, thus the correct classification of various inks is strongly related to the real differences in chemical composition between the inks, and the same compared to the paper materials included in the data set. The reduced classification rate when different paper types are considered is not surprising in document examination where casework items are always examined by multiple techniques in sequence to maximize the discrimination power of the overall forensic procedure.

### 3.3. Characterization of Inks on Cross Points

The LIBS analysis on the sample type shown in Figure 1a was conducted in two steps. Firstly, the preliminary studies were performed on a duplicate sample, where the measurements were performed at six points per ink and on blank paper in order to estimate the fluctuation of the peak intensities; the corresponding results are given in Table 6. The paper itself did not show the presence of these elements. The ink used to draw the arrow contained only Cu, Zn and a very low amount of Pb, but its Cu content was at least three times lower than in the other three inks. Ink 1 had the highest contents of Cu, Zn, and Pb among the samples while Mn and Ni were not detected. Inks #2 and #3 had very similar compositions and contained all five metals, but they could be differentiated through much larger presence of Mn in ink #3.

On a different day, the subsequent laser probing was done on the second sample in conditions for minimal damage, as required in true forensic analysis. Here, the LIBS measurements were performed in a single spot per four inks and three crosses. For the inks, the obtained values of the characteristic element peaks remained inside the errors reported in Table 6.

**Figure 7:**
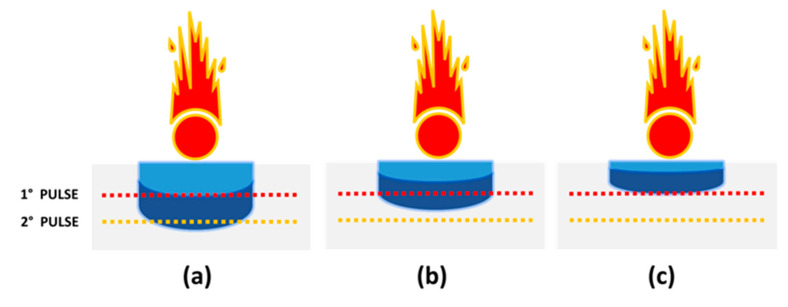
Illustrated laser ablation of two overlapped inks: (**a**) ideal case for LIBS analysis; (**b**) partial ablation of the underlying ink; (**c**) both inks are simultaneously ablated.

Because LIBS detects elements originally present in the ablated layer, in an ideal case for distinguishing the deposition order of two inks their mixing is absent and the ablation rate is smaller than the thickness of the top ink layer (Figure 7a). Differently, the spectra acquired sequentially at a cross point do not fully reflect the ink compositions in the last pen stroke (above) if the first laser pulse also partially ablates the ink beneath (Figure 7b) e.g., when the layer of the ink due to the last pen stroke is too thin or when the inks are partially mixed. Finally, if the two ink layers are too thin compared to the ablation depth, it is impossible to discriminate their deposition order using LIBS (Figure 7c). Reducing the laser pulse energy, i.e., the ablation rate per pulse, leads to less intense plasma emission and consequent loss of information about the minor or trace elements in the sample.

**Figure 8:**
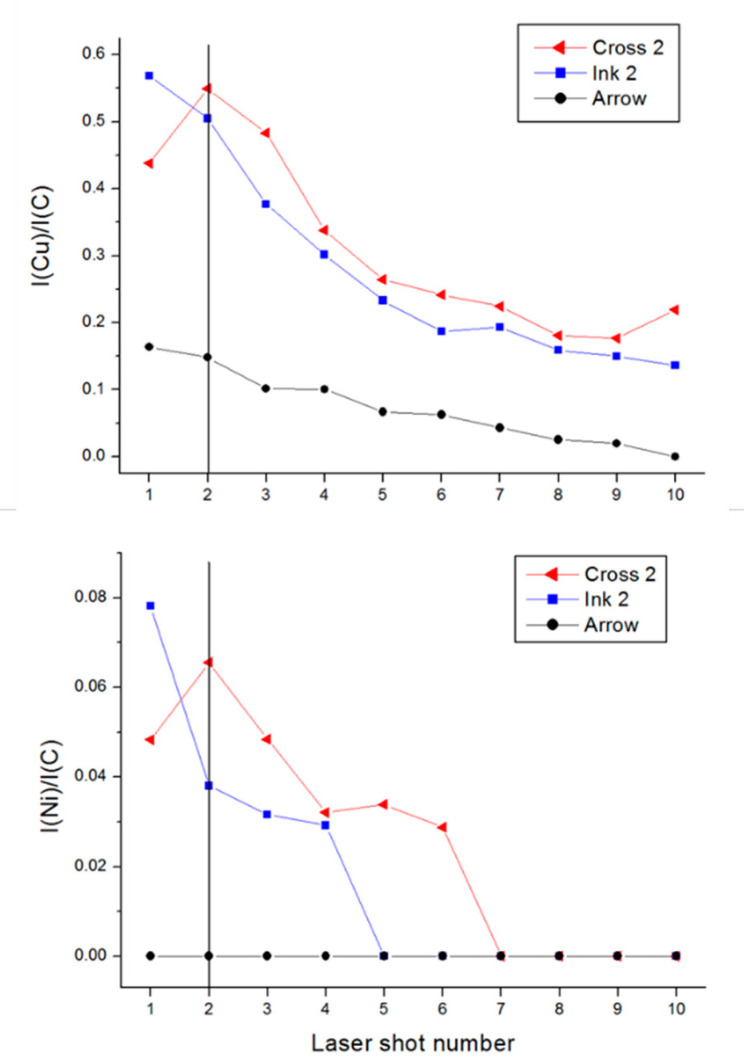
Normalized peak intensity of Cu (**top**) and Ni (**bottom**) as a function of the laser pulse number during the profiling of cross 2; the vertical line indicates the dominant position of Ink 2.

Figure 8 shows an example of the normalized Cu I and Ni I line intensities during the depth profiling of cross 2 (central cross in Figure 1a). On a single ink, the normalized intensities from the characteristic elements (Cu/C, Ni/C, etc.) usually decrease with the sequential laser pulses, where the decay rate also depends on the absorbing properties of the paper (Figure 5), the local line thickness and the ink density. In Figure 8, the Cu/C content is much higher on Ink 1 than on arrow, the latter does not contain Ni. The differences in decay rates with the shot number among the detected elements might arise from different element penetration depths into the paper and from changes of the plasma parameters from one shot to another, which affect the intensities of the analytical lines dependently on their excitation level and on the atom-to-ion ratio in the plasma. At the cross point the first laser pulse produced Cu/C and Ni/C values between those obtained on the pen strokes far from the crossing point, meaning that also the first deposited layer was partially ablated, as illustrated in Figure 7b. These ratios show a rise after the second pulse, indicating that the deeper layer contains higher amounts of these elements; in the specific case, this means that the arrow was placed above ink 2.

Therefore, we considered the ratio R of the normalized line intensities after the first and the second laser shot as a possible parameter for discriminating the deposition order (Table 7). With the aim to reduce the data scattering for Cu, here we considered a weighted sum of the peaks from the Cu I line and Cu II transition at 224.70 nm. From Table 7 it is evident that the measurements on a single ink lead to a decay of the normalized peak intensities during the first two pulse numbers; this measured change was higher for Pb and Ni compared to Cu. For the cross points we considered the peak change with the shot number to be significant if it reaches at least 10% (R ≤ 0.9 or R ≥ 1.1). Based on this threshold, it results that on cross 1 for measure 2, the top ink contains less Pb, i.e., the arrow was placed over ink 1. For measurement m1, the detected differences were insufficient to establish the deposition order. On cross 2, the first measurement identified lower contents of Cu and Ni in the top layer, which were attributed to the arrow; the analogue changes in measurement 2 were too small to be conclusive. For cross 3, both measurements showed a large increase of Cu content from the first to the second laser shot, indicating that ink 3, which is rich in this element, had been deposited first.

**Figure 9:**
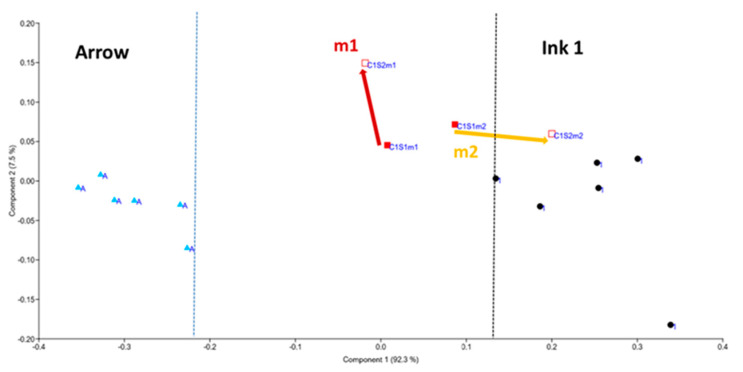
PCA plot for cross 1, considering Cu/C, Pb/C, and Zn/C values.

The proposed method for establishing the deposition order of inks supplied the correct answer on the blind test in four cases out of six, and no answer in the remaining two cases. Differently from the tested cases, this approach might be difficult to apply if an ink rich in metals is deposited over an ink with low metal contents because the signal decay with the laser pulse number was always observed on single inks. To overcome this potential problem, we applied PCA analysis on each pair of inks, probed six times, and on the corresponding crosses; the example regarding cross 1 is shown in Figure 9. Here, it might be observed that the two inks are well separated. The first measurement on the cross produces the values in between, both after the first and the second laser pulse, meaning that the two layers were too thin compared to the laser ablation rate and could not be discerned (see Figure 7c). In the second measurement, the first laser pulse partially ablated also the underlying ink (Figure 7b), and the corresponding point is in between the two inks. The second laser pulse reached the area in the plot that is characteristic for ink 1, leading to the conclusion that the arrow was deposited on the top. The analogue PCA plot for cross 2 and considering Cu/C, Pb/C and Ni/C, in the first measurement indicated that the arrow was placed over ink 2. In the second measurement, for both laser pulses the points in the PCA plot fell in the area of ink 2, meaning that the layer left by the arrow was locally very thin and placed on the top. This hypothesis is enforced by the point moving in direction from the arrow (shot 1) to ink 2 (shot 2). On cross 3, both measurements indicated that the first laser pulse ablated the arrow and partially ablated ink 3 while the second laser pulse brought to the composition of ink 3, which was placed first on the paper.

Finally, in the blind test where the arrow was placed over the other three lines (inks), the approach based on the ratio of peak intensities after the first and second laser pulses correctly identified the deposition order in four cases out of six; the remaining two cases were inconclusive. One more case was identified correctly by using PCA analysis while the one missing answer could be attributed to locally very thin ink layers compared to the laser ablation depth.

### 3.4. Characterization of Inks and Toners on an Questioned Document

**Figure 10:**
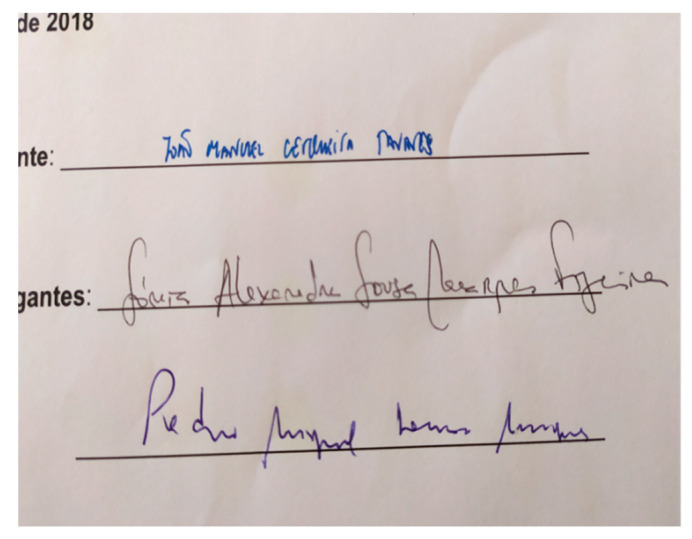
Photograph of the questioned document - part containing the final extended signatures.

In order to further evaluate the capability of LIBS for forensic applications, we participated in a blind test on a questioned document provided by the Portuguese Police Forensic Laboratory. The questioned document was a contract containing three printed pages; at each page there were three different signatures, clearly made by three different pens—one light blue, one dark blue, and one black (Figure 10). The goal was to determine whether the document had been printed by same ink or toner and whether the signatures were made by the same ink on every page.

With the aim of minimizing damage, the depth profiling by LIBS was performed at a single point on each signature, using ten laser pulses delivered in succession. The comparative measurements on the blank paper were performed at six points close to the sheet’s edges and not affected by any ink. We found that the light blue signatures are rich in Cu, not found in the paper. The measured Cu/C line peak ratio did not differ significantly among the three pages. However, the signature on page two is different because it also contains Mn, absent on the other two pages; its spectrum showed a number of relatively intense Ba II lines, not observed on the corresponding paper or on the other two light blue inks. From the LIBS spectra we found that the dark blue signatures contain a high amount of copper. The ink on page #2 is different, as it has about a two-fold higher Zn/C ratio than at pages 1 and 3, and also contains Fe, as observed from different atomic and ionic lines. This ink on page 2 probably also contains Li and K (Table 8), but these two elements were also present in the paper and their normalized peaks strongly fluctuated from one measuring point to another, so their attribution to the inks requires some caution. The black signatures contain a small amount of copper with similar Cu/C values observed among the three pages. However, for the signatures on pages 1 and 3 we observed the Pb I line in the spectrum, which was slightly above the detection threshold, while on page 2 the spectrum after the first pulse showed a weak Zn II peak. These observations imply that the black ink on page 2 is probably different, but this conclusion is based on very low discriminatory signals (from Pb and Zn) and single point measurements.

The LIBS sampling on toners was performed at six points per page. From the comparative measurements on the toners and the papers, we found that the printed layer was thick enough to effectively screen the substrate during ablation by the first laser pulse; the LIBS spectra from papers exhibited relatively intense lines from Al I, Sr I, and Li I while these lines were very weak or absent when acquiring the signal on the toners (by the first laser pulse). The characteristic elements found in the toners are reported in Table 9 as normalized peak values. The measured values of K/C were much larger in toners than on the corresponding papers while the low presence of Ca and Mg in the spectra might be due to residual ablation of the underneath paper. From Table 9 it is evident that the toner on page 2 is different because it does not contain Ti, Ca, or Mg while the presence of Si is much higher than in the other two cases. The toners on pages 1 and 3 contain the same relative amounts of Si, Ti, Ca, Mg, and K (within the measuring error). The last three elements show a large variability from one measuring point to another, as previously observed for inks and blank paper samples, so their use for classification of toners or inks must be taken with caution.

The blind test by LIBS on the questioned document was successful, as it revealed that the signatures on page 2 are different from those on the other two pages, and that page 2 was printed by a different toner.

## 4. Conclusions

The experimental work here demonstrated that LIBS measurements using the portable apparatus allow the questioned documents to be analyzed without any sample preparation and with minimal damage. The examined pen inks used to write on paper showed different contents of seven metals (Cu, Zn, Mn, Ni, Pb, Fe, and Cr), but their spectral peaks, although normalized on carbon, varied significantly from one point to another due to the locally variable thickness of the ink layer, which is dependent on the exerted pressure and speed during writing. However, these analyses already tag the differences among the ink elemental compositions.

A preliminary PCA analysis, involving the normalized peaks of the selected elements, allowed the discrimination of all eight black inks, producing 100% correct attribution by LDA classifier. When studying the same inks on different types of papers, difficulties in their discrimination arise. One reason is the presence of the same elements in both the ink and the paper ablated simultaneously with ink. Another reason is the different penetration of inks into paper, increasing the possible variability of the analytical results in the casework. In case of a highly absorbing paper, the LIBS signal from the characteristic ink elements after the first laser pulse is weaker compared to a case of less permeable paper, but the signal persists for a larger number of subsequent laser pulses. As a result, the data points on the PCA plots are much more scattered than in case of tests on one paper, and the LDA classifier on ten different paper types produced 100% correct results for only four inks out of eight. Erroneous attribution (11% overall) was particularly evident for the inks containing elements important for differentiation in traces, which are present also in some papers.

Another interesting result of our research was obtained from the third group of tests, by applying LIBS depth profiling on points where two lines, made of different inks, cross: it was possible to establish correctly the order of layering of two inks in five cases out of six. In one case, it was impossible to provide the answer, probably because of locally very thin ink layers compared to the laser ablation depth. The elements of the secondly placed ink were present in the spectrum acquired by the first laser pulse and disappeared for the following pulses. The elements of the firstly placed ink emerged after the second laser pulse delivered to the crossing point and were also present in the successively acquired spectra before the unaltered paper layer was reached.

In the fourth group of tests, it was possible to demonstrate by single point LIBS analysis the chemical differences in three inks employed for signatures at one of the three pages. Moreover, the repeated LIBS measurements carried out on both printing toners and the papers, correctly discriminated the toners basing on relative amounts of Si, Ti, Ca, Mg, and K.

We can, therefore, conclude that the application of the LIBS technique coupled with multivariate statistical tools and comparison methods based on the content of specific elements, is an easy and useful tool for distinguishing inks and toners with different elemental compositions. The developed method is rapid, it does not require sample preparation, it does not destroy the analyzed item, and it does not require initial calibration. We are perfectly aware that a single analytical technique is rarely able to supply sufficient information for forensic casework, but we expect a more widespread exploitation of LIBS in forensic procedures for document examination.

## Figures and Tables

**Table 1:** Inks from commercial ballpoint pens that were analyzed and the characteristic elements detected by Laser-induced breakdown spectroscopy (LIBS).

N°	Brand/Producer	Type/Model	Ink Color
**1**	Papermate	Flexgrip Ultra 1M	**Black**
**2**	Tratto	Uno 1	**Black**
**3**	Pilot	BP-S Matic Fine	**Black**
**4**	Bic	Soft Feel Med	**Black**
**5**	Staedtler	Noris Stick	**Black**
**6**	Papermate	Inkjoy 300 RT 1M	**Black**
**7**	Pilot	G-1 Grip	**Black**
**8**	Faber Castell	Lux 300	**Black**
**9**	Papermate	Flexgrip Ultra 1M	**Blue**
**10**	Tratto	Uno 1	**Blue**
**11**	Pilot	G-1 Grip	**Blue**
**12**	Bic	Cristal	**Blue**
**13**	Bic	Cristal	**Red**
**14**	Bic	Cristal	**Green**

**Table 2:** Full specifications for the questioned document.

	Lease Agreement	
	Pages 1 and 3	Page 2
**Paper**	Inacópia office 80 g/m^2^, 100 µm	Staples 80 g/m^2^, 100 µm
**Printer**	Konica Minolta model bizhub	OKI Model ES8453 MFP223
**Wrinting inks**	*Signature of the first grantor*	
	Blue Gel pen Mitsubishi uni.ball Signo	Blue Gel pen Mitsubishi uni.ball Signo 0.7
	*Signature of the second grantor*	
	Black ballpoint pen BIC	Black ballpoint pen Pentel SUPERB
	*Signature of the third grantor*	
	Blue ballpoint pen “white label”	Blue ballpoint pen OfficeCover Astro 1.0

**Table 3:** The considered element lines and their presence in the spectra from the blank paper.

Element	State *	Wavelength (nm)	Paper
Al	I	309.271	+
Ba	II	455.403	-
C	I	247.856	+
Ca	I	452.69	+
Cr	I	425.222	-
Cu	I	324.754	-
Fe	II	238.204	-
H	I	656.279	+
K	I	769.896	+
Li	I	670.776	+
Mg	I	285.213	+
Mn	II	257.610	-
Na	I	589.592	+
Ni	I	352.454	-
Pb	I	405.781	-
Si	I	288.158	+
Sr	II	407.771	+
Ti	I	498.173	-
Zn	II	202.548	-

* I = atomic, II = first ionization.

**Table 4:** Line intensities of the characteristic ink elements normalized on the C I line in the spectra taken after the first laser pulse. The values were determined for the inks placed on printing paper. The first column (No) is the ink number.

No	Cu/C	Zn/C	Mn/C	Ni/C	Pb/C	Fe/C	Cr/C
1	0.92 ± 0.22	0.16 ± 0.09	0.27 ± 0.11	0.11 ± 0.02	0.033 ± 0.006	0.017 ± 0.004	0
2	0.056 ± 0.009	0	0	0.051 ± 0.011	0	0	0
3	0	0	0.078 ± 0.013	0	0	0	0.50 ± 0.24
4	2.86 ± 0.78	0.41 ± 0.07	0	0.0304 ± 0.009	0.078 ± 0.035	0	0
5	1.56 ± 0.60	0.20 ± 0.08	0	0.033 ± 0.003	0.028 ± 0.005	0	0
6	0.39 ± 0.04	0.089 ± 0.013	0	0.061 ± 0.013	0	0	0
7	0	0	0.064 ± 0.015	0	0	0	0
8	1.00 ± 0.33	0.20 ± 0.07	0	0	0.028 ± 0.011	0	0.029 ± 0.005
9	0.75 ± 0.31	0.13 ± 0.05	0.22 ± 0.09	0.098 ± 0.044	0.041 ± 0.014	0.023 ± 0.012	0
10	0.28 ± 0.02	0.046 ± 0.001	0.10 ± 0.02	0.059 ± 0.03	0.017 ± 0.006	0.019 ± 0.008	0
11	0.043 ± 0.002	0	0.078 ± 0.007	0	0	0.012 ± 0.005	0
12	3.5 ± 1.0	0.36 ± 0.10	0	0.053 ± 0.02	0.058 ± 0.004	0	0
13	3.0 ± 1.0	0.29 ± 0.09	0	0.042 ± 0.007	0.091 ± 0.020	0.022 ± 0.010	0
14	2.7 ± 1.2	0.29 ± 0.07	0	0.046 ± 0.011	0.039 ± 0.009	0.013 ± 0.005	0

**Table 5:** Confusion matrix for linear discriminant analysis (LDA) classification algorithm applied on eight black inks placed over ten different papers (six measuring points for each paper–ink combination).

	1	2	3	4	5	6	7	8	Total
**1**	**60**	0	0	0	0	0	0	0	60
**2**	0	**60**	0	0	0	0	0	0	60
**3**	0	0	**60**	0	0	0	0	0	60
**4**	0	0	0	**48**	4	2	0	6	60
**5**	0	0	0	0	**50**	10	0	0	60
**6**	0	20	0	0	1	**39**	0	0	60
**7**	0	0	0	0	0	0	**60**	0	60
**8**	0	0	0	4	2	0	0	**54**	60
**Total**	60	80	60	52	57	51	60	60	**480**

**Table 6:** Values of the normalized peaks of the characteristic element lines obtained after the first laser pulse on four inks and the paper prior to analysis of the cross points.

Ink	Cu/C	Zn/C	Mn/C	Ni/C	Pb/C
ARROW	**0.157 ± 0.038**	0.072 ± 0.014	0	0	0.035 ± 0.008
INK 1	0.788 ± 0.162	**0.105 ± 0.014**	0	0	**0.078 ± 0.005**
INK 2	0.606 ± 0.119	0.054 ± 0.009	0.054 ± 0.009	0.086 ± 0.025	0.046 ± 0.005
INK 3	0.572 ± 0.105	0.073 ± 0.008	**0.145 ± 0.011**	0.077 ± 0.016	0.042 ± 0.009

**Table 7:** Ratio R of the normalized line peaks after the first and second laser pulses, and the results of the PCA analysis where → indicates the shift in the composition from the first to second pulse; m1 = measurement 1, m2 = measurement 2.

Sample	R(Cu)	R(Pb)	R(Ni)	PCA
Ink 1	1.22 ± 0.19	1.50 ± 0.27	-	
Ink 2	1.15 ± 0.15	1.37 ± 0.23	1.47 ± 0.38	
Ink 3	1.15 ± 0.18	1.50 ± 0.31	2.12 ± 0.61	
Arrow	1.09 ± 0.07	1.31 ± 0.13	-	
Cross 1-m1	0.97	1.06		Mixed layers
Cross 1-m2	1.02	0.86 ^+^		Arrow → Ink 1
Cross 2-m1	0.90 ^+^		0.74 ^+^	Arrow → Ink 2
Cross 2-m2	1.05		1.01	Arrow → Ink 2
Cross 3-m1	0.85 ^+^		1.06	Arrow → Ink 3
Cross 3-m2	0.81 ^+^		0.92	Arrow → Ink 3

^+^ Deposition order is determined.

**Table 8:** Characteristic line peak ratios for discriminating the dark blue signatures by LIBS after applying the first laser pulse. The relatively weak Zn line was fitted by the Gaussian function.

Line Peak Ratio	Sample	Page 1	Page 2	Page 3
Cu/C	Ink	0.45	0.54	0.33
	Paper	0	0	0
Zn/C	Ink	0.012	**0.026**	0.013
	Paper	0	0	0
Fe/C	Ink	0	**0.018**	0
	Paper	0	0	0
Li/C	Ink	0.020	**0.11**	0.018
	Paper	0.025 ± 0.016	0.052 ± 0.020	0.025 ± 0.028
K/C	Ink	0.088	**0.51**	0.17
	Paper	0.11 ± 0.02	0.19 ± 0.11	0.12 ± 0.04

**Table 9:** Normalized peaks of the analytical lines of the elements characteristic for the printing inks/toners on the questioned document.

Page	Si/C	Ti/C	Ca/C	Mg/C	K/C
1	0.12 ± 0.03	0.43 ± 0.07	0.05 ± 0.03	0.22 ± 0.12	0.27 ± 0.05
2	0.47 ± 0.09	0	0	0	0.27 ± 0.13
3	0.11 ± 0.02	0.38 ± 0.05	0.06 ± 0.02	0.18 ± 0.10	0.37 ± 0.14

## References

[1] Calcerrada M., García-Ruiz C. (2015). Analysis of questioned documents: A review. Anal. Chim. Acta.

[2] Denman J.A., Skinner W.M., Kirkbride K.P., Kempson I.M. (2010). Organic and inorganic discrimination of ballpoint pen inks by ToF-SIMS and multivariate statistics. Appl. Surf. Sci..

[3] Ezcurra M., Góngora J.M.G., Maguregui I., Alonso R. (2010). Analytical methods for dating modern writing instrument inks on paper. Forensic Sci. Int..

[4] Gorziza R.P., Bello de Carvalho C.M., González M., Leal L.B., Korndörfer T., Ortiz R.S., Trejos T., Limberger R.P. (2019). Blue and black ballpoint pen inks: A systematic review for ink characterization and dating analysis. Braz. J. Forensic Sci. Med. Law Bioeth..

[5] Neumann C., Ramotowski R., Genessay T. (2011). Forensic examination of ink by high-performance thin layer chromatography, The United States Secret Service Digital Ink Library. J. Chromatogr. A.

[6] Saini K., Rathore R. (2018). Differentiation of Red and Black Ballpoint Pen Inks using High Performance Thin Layer Chromatography and Gas Chromatography-Mass Spectrometry. Arab J. Forensic Sci. Forensic Med..

[7] Alamilla F., Calcerrada M., García-Ruiz C., Torre M. (2013). Forensic discrimination of blue ballpoint pens on documents by laser ablation inductively coupled plasma mass spectrometry and multivariate analysis. Forensic Sci. Int..

[8] Trejos T., Flores A., Almirall J.R. (2010). Micro-spectrochemical analysis of document paper and gel inks by laser ablation inductively coupled plasma mass spectrometry and laser induced breakdown spectroscopy. Spectrochim. Acta Part B.

[9] Agarwal A., Sharma N., Negi Y.S. (2016). Review: Techniques for the characterization of inks. IOSR J. Appl. Chem..

[10] Braz A., López-López M., Garcì-Ruiz C. (2013). Raman spectroscopy for forensic analysis of inks in questioned documents. Forensic Sci. Int..

[11] Silva C.S., Braz A., Pimentel M.F. (2019). Vibrational Spectroscopy and Chemometrics in Forensic Chemistry: Critical Review, Current Trends and Challenges. J. Braz. Chem. Soc..

[12] Zieba-Palus J., Borusiewicz R., Kunicki M. (2008). PRAXIS--combined μRaman and μXRF spectrometers in the examination of forensic samples. Forensic Sci. Int..

[13] Claybourn M., Ansell M. (2000). Using Raman spectroscopy to solve crimes: Inks questioned documents and fraud. Sci. Justice.

[14] Mazzella W., Khanmy-Vital A. (2003). A study to investigate the evidential value of blue gel pens inks. J. Forensic Sci..

[15] Buzzini P., Polston C., Schackmuth M. (2018). On the criteria for the discrimination of inkjet printer inks using micro-Raman spectroscopy. J. Raman Spectrosc..

[16] Braz A., López-López M., García-Ruiz C. (2014). Studying the variability in the Raman signature of writing pen inks. Forensic Sci. Int..

[17] Silva C.S., Borba F.S.L., Pimentel M.F., Pontes M.J.C., Honorato R.S., Pasquini C. (2013). Classification of blue pen ink using infrared spectroscopy and linear discriminant analysis. Microchem. J..

[18] Sharma V., Kumar R. (2017). Fourier transform infrared spectroscopy and high-performance thin layer chromatography for characterization and multivariate discrimination of blue ballpoint pen ink for forensic applications. Vib. Spectrosc..

[19] Sharif M., Batool M., Chand S., Farooqi Z.H., Tirmazi S.A.A.S., Athar M. (2019). Forensic discrimination potential of Blue, Black, Green, and Red colored fountain pen inks commercially used in Pakistan, by UV/Visible spectroscopy, thin layer chromatography, and Fourier transform infrared spectroscopy. Int. J. Anal. Chem..

[20] Armstrong C., Brown J.F., Mackenzie M.J., Randall L., Smith H.G.B. (2012). The Printing Ink Manual.

[21] Materazzi S., Risoluti R., Pinci S., Romolo F.S. (2017). New insights in forensic chemistry: NIR/Chemometrics analysis of toners for questioned documents examination. Talanta.

[22] Król M., Karoly A., Kóscielniak P. (2014). Raman spectroscopy and capillary electrophoresis applied to forensic colour inkjet printer inks analysis. Forensic Sci. Int..

[23] Johnson C.E., Martin P., Roberts K.A., Trejos T., Corzo R., Almirall J.R., Safer A.M. (2018). The capability of Raman microspectroscopy to differentiate printing inks. J. Forensic Sci..

[24] Almeida Assis A.C., Barbosa M.F., Valente Nabais J.M., Custódio A.F., Tropecelo P. (2012). Diamond cell Fourier transform infrared spectroscopy transmittance analysis of black toners on questioned documents. Forensic Sci. Int..

[25] Lennard C., El-Deftar M.M., Robertson J. (2015). Forensic application of laser-induced breakdown spectroscopy for the discrimination of questioned documents. Forensic Sci. Int..

[26] Hoehse M., Paul A., Gornushkin I., Panne U. (2012). Multivariate classification of pigments and inks using combined Raman spectroscopy and LIBS. Anal. Bioanal. Chem..

[27] Kula A., Wietecha-Posłuszny R., Pasionek K., Król M., Woźniakiewicz M., Kościelniak P. (2014). Application of laser induced breakdown spectroscopy to examination of writing inks for forensic purposes. Sci. Justice.

[28] Elsherbiny N., Nassef O.A. (2015). Wavelength dependence of laser induced breakdown spectroscopy (LIBS) on questioned document investigation. Sci. Justice.

[29] Rzecki K., Sośnicki T., Baran M., Niedźwiecki M., Król M., Łojewski T., Acharya U.R., Yildirim Ö., Pławiak P. (2018). Application of computational intelligence methods for the automated identification of Paper-Ink samples based on LIBS. Sensors.

[30] Subedi K., Trejos T., Almirall J. (2015). Forensic analysis of printing inks using tandem Laser Induced Breakdown Spectroscopy and Laser Ablation Inductively Coupled Plasma Mass Spectrometry. Spectrochim. Acta Part B.

[31] Metzinger A., Rajkó R., Galbács G. (2014). Discrimination of paper and print types based on their laser induced breakdown spectra. Spectrochim. Acta Part B.

[32] Hui Y.W., Mahat N.A., Ismail D., Ibrahim R.K.R. (2019). Laser-induced breakdown spectroscopy (LIBS) for printing ink analysis coupled with principle component analysis (PCA). AIP Conf. Proc.

[33] Oujja M., Vila A., Rebollar E., Garcìa J.F., Castillejo M. (2005). Identification of inks and structural characterization of contemporary artistic prints by laser-induced breakdown spectroscopy. Spectrochim. Acta Part B.

[34] Brito L.R., Martins A.R., Braz A., Chaves A.B., Braga J.W., Pimentel M.F. (2017). Critical review and trends in forensic investigations of crossing ink lines. Trends Analyt. Chem..

[35] Goacher R.E., DiFonzo L.G., Lesko K.C. (2017). Challenges determining the correct deposition order of different intersecting black inks by Time-of-Flight secondary ion mass spectrometry. Anal. Chem.

[36] Li B., Ouyang G., Zhao P. (2018). Preliminary study on determining the sequence of intersecting lines by fluorescence technique. J. Forensic Sci..

[37] Malloy M., Bogdanović Radović I., Siketić Z., Jakšić M. (2018). Determination of deposition order of blue ballpoint pen lines by MeV SIMS. Forensic Chem..

[38] Brito L.R., Chaves A.B., Braz A., Pimentel M.F. (2019). Raman hyperspectral imaging and a novel approach for objective determination of the order of crossing ink lines. Spectrochim. Acta Part A.

[39] Lazic V., De Ninno A. (2017). Calibration approach for extremely variable laser induced plasmas and a strategy to reduce the matrix effect in general. Spectrochim. Acta Part B.

[40] Amador V.S., Pereira H.V., Sena M.M., Augusti R., Piccin E. (2017). Paper spray mass spectrometry for the forensic analysis of black ballpoint pen inks. J. Am. Soc. Mass Spectrom.

[41] Williams M.R., Moody C., Arceneaux L.A., Rinke C., White K., Sigman M.E. (2009). Analysis of black writing ink by electrospray ionization mass spectrometry. Forensic Sci. Int..

